# Supplementary material for: Quality of life and metabolic outcomes after total pancreatectomy and simultaneous islet autotransplantation
Source: Commun Med (Lond). 2022 Mar 3;2:24. doi: 10.1038/s43856-022-00087-7 (PMC9053265; doi:10.1038/s43856-022-00087-7)
Supplement: Supplementary file 4 — Description of Additional Supplementary Files [file 43856_2022_87_MOESM4_ESM.pdf]

Description of Additional Supplementary Files

File Name: Supplementary Data 1

Description: The datasets generated during and/or analysed during the current study
